# The Gingival Third

**Published:** 1911-04-15

**Authors:** J. V. Conzett

**Affiliations:** Dubuque, Iowa


### THE GINGIVAL THIRD.

J. V. CONZETT, DUBUQUE, IOWA.

Read before the Missouri State Dental Association, 1910.

There are more pathological disturbances in and around the gingival third of the tooth than in all the other surfaces of the teeth combined. By that I do not mean that decay more frequently takes place at this region than at others, for such is not the fact, but I do mean that this surface and its immediate environment is the seat of more diseases than the other surfaces of the teeth. We not only have caries, and in its most difficult form, but we also have gingivitis, the beginning of so-called pyhorrhea, erosions, both chemical and abrasive, and a condition that is probably a beginning of caries, but its primary manifestation is an extreme sensitiveness at the gingival margin.

It is not my intention to attempt the discussion of pyorrhea at this time, important as I deem that subject to be, but the scope of this paper will be too wide as it is without attempting any discussion of the diseases of the gingivae. Neither will I discuss the subject of erosion, but will confine my efforts to an attempt to bring before you some phases of the beginnings of caries and their treatment.

We frequently find a patient presenting with a complaint that the "necks" of their teeth are excruciatingly tender and that they pain severely at the slightest touch and are sensitive to the cold and sweets. Ocular examination reveals no apparent lesion, but at the slightest touch of the explorer the patient almost jumps out of the chair; there is no decay, at least it is not demonstrable to macroscopical examination, although from clinical experience I believe that it would be demonstrated microscopically. The question is what will we do with these cases? The patient demands some relief and it is our duty to give them the relief that they demand or the teeth will be lost or the patient will be lost to us.

Recently I have had three cases and it is this that has influenced me to bring this matter to your attention. The first case was that of a lady forty-five years old, who had just had an operation for appendicitis, which had been followed by a post-operative pneumonia. During the course of her convalescence she complained very much of her teeth hurting her, the "necks," as she expressed it, being so sensitive that she suffered severely; any attempt to drink cold water or eat any sweet caused so violent a pain that she refused to partake of anything cold or sweet, and the use of the tooth brush was so severe that she was forced to discontinue its use. She appealed to the physicians and the nurses for relief, but was assured that nothing could be done for her, and so without the attendance of a dentist she was allowed to suffer through the period of her convalescence until she was discharged and went home, when as soon as possible, I was called in and in a few minutes gave her so much relief that she begged me to let the medical profession know that such relief could be obtained that no one else should be made to suffer as she had suffered. Of course what I did is what any dentist could and would have done, that is, the sensitive places at the gingival margin were treated with a saturated solution of the nitrate of silver and so exposed to the light that the silver salts were deposited within the slightly decalcified dentinal tubules and the result was immediate relief. The second case was like unto the first, only the predisposing cause was not a physical ailment, but rather one of a mental, or perhaps I might better say, a nervous disease. The daughter of a patient suddenly contracted a peculiarly distressing malady which so worried the mother that she was almost driven into nervous prostration. After a time she presented with the same train of symptoms as the first patient, and was in such a condition that only the most casual treatment was possible. The nitrate of silver treatment was instituted with the same happy result. And now, since the inception of this paper, has come the third case within a comparatively short time, and under circumstances that make the train of cases highly interesting if not positively suggestive. The third case is that of a man who had a severe fall. He was cleaning the snow off of his house and fell and in falling fell on his back on a picket fence. It was at first thought that he had broken his back, but fortunately such was not the case, but instead he suffered and is still suffering from a severely sprained back. The dental result was identical with the other causes reported, and the treatment the same, nitrate of silver method.

The suggestive thing in all of these cases was the immediate following of a severe physical disturbance by this same dental disease. In all of these cases I am convinced that the nervous condition played a very large part of the dental result, for all of the patients were of a decidedly neurotic temperament, which was emphasized by the exigencies of the disease. The question that naturally arises is, is there a secretion from the gingivae at such periods that will have a decalcifying influence at the gingival margin at this time, or is the period of such stress for some reason or other a period of susceptibility and the failure to keep these margins scrupulously clean caused the deposition of the bacterial placques that eventually brought about the result? While I am a firm believer in the bacterial theory of Williams, Miller and Black, I am inclined to the belief that in these cases the primary effect, at least, is brought about by some vitiated secretion of the mucous follicles in the gingival region. My reason for such a belief is that in two of these cases I had had the constant supervision of the patients' teeth and knew that the teeth immediately prior to the operation and the nervous break down, were in perfect condition and in all of the cases there was no evidence of any other decay in any of the other teeth. And yet while there was no decay anywhere else, the gingival margin of all of the ten anterior upper teeth were affected. If it was not a local but a general cause, why in all three of these patients did we have the localized effect and no general decay?

The subsequent history of the two first cases may help us a little. The teeth were kept in a comfortable condition although there was a constantly growing area of blackened tissue as a result of the continued use of the silver nitrate necessitated by the recurring sensitiveness of the teeth until the time came when it was possible to cut out the decay which was now very apparent, and fill the teeth, which was accomplished with the porcelain inlay for the cosmetic effect. There was no doubt about the decay when the teeth were filled and as they were constantly under the influence of the silver nitrate, the question may be raised as to the possibility of the organisms of decay operating under such circumstances. I don't know; I leave that for a wiser head than I have to determine.

While the cases enumerated were unquestionably exaggerated cases, there are many cases that present for treatment that call just as loudly for relief and in the majority of cases they can be permanently relieved and the decalcifying process stopped. This may be dome by the use of the silver nitrate as suggested, but I prefer to use the high speed burnisher method first. This is done by placing a smooth ball burnisher in the engine and then rapidly and firmly go over the sensitive surface of the tooth. It will cause considerable pain at first, but as you progress the pain will become more bearable and will finally cease, or if not will be very much better and at a subsequent treatment the sensitiveness will be almost, if not entirely, relieved. I prefer to try this method first because it does not discolor the tooth and if it does relieve the sensitiveness, the progress of the disease or decay can be stopped by the institution of prophylaxis on the part of the dentist and scrupulous care on the part of the patient in brushing the teeth. A favorite wash for use on the brush at such times has been a teaspoonful of salt and a teaspoon of soda bicarbonate to the pint of water, but the suggestion has recently been made to brush the teeth with the bicarbonate of soda as a dentifrice. In these cases a little caution is necessary in brushing the teeth. While it is necessary to keep the surfaces of the teeth scupulously clean it is not well to use an abrasive in such a way that the slightly softened tissue at the gingival region will be cut away and a decided mechanical abrasion follow. The patient should be instructed to brush the teeth with a motion that will carry the brush from the gingival to the occlusal surface in both jaws. That is, brush the upper teeth down and the lower teeth up. Dr. Black advises the use of no dentifrice at all in these cases, only insist upon the careful and frequent brushing of the surfaces with clean water.

We often find the beginnings of decalcification in these regions made manifest by a white line in the enamel, and if immediate methods are not put into operation to combat the process the teeth will decay along the line of the white area. If the enamel is not broken it is possible to stop these processes in many cases. But the dentist, in order to do so, must have the cooperation of the patient, for no matter how carefully the dentist cleanses these surfaces, unless the patient does his share in the interim between treatments, the efforts of the dentist will fail, and the patient should be so instructed. These surfaces, being plain surfaces, are easily kept clean and the progress of decay is, therefore easily prevented. In these cases the dentist should insist upon the regular presentation of the patient for prophylactic treatment. It is my practice to urge such treatment upon all of my patients, with the happiest results to both patient and dentist. Several years ago a lady presented with the gingival thirds of nearly all of her teeth showing decided evidences of the inception of caries in that there was a decided white line of beginning decalcification in this neighborhood. The condition was pointed out to her and as she was already under my care at regular intervals, these periods were shortened and she was requested to come at more frequent intervals. The result has been that now, after two years of such treatment, there has been no further progress of the decalcification and in fact the white areas are growing smaller at each treatment.

When the decay has penetrated the enamel there is no choice in the matter but that the decay must be cut out and the cavity filled. For it is a fact beyond question that as soon as the decay reaches the dentine that decay is rapid and practically unpreventable. The only question now is what to fill with and how to do it.

Gold is the surest and most permanent material that we have with which to fill teeth, and in those cases that do not call for the cosmetic effect first, and the permanency of the result as a secondary consideration, is the material that should be selected. But I realize that we cannot treat our patients as though they were inanimate objects and so we must select the material and the method that the conditions demand.

In filling these cavities with gold it is necessary to use the rubber dam, and that calls for a clamp of some kind, as the location of the decay demands the retraction of the gingivae so that the cavity may be cut far enough to carry the margin under the gum, for if we do not do so we invite a recurrence of decay at some future time. There are two clamps that may be used with success, the Hatch clamp and the Ivory Gingival clamp, with the preference in favor of the Ivory, in my opinion.

After the dam is adjusted and the surface of the tooth carefully wiped off with alcohol, the cavity is prepared with a flat axial wall (which is prepared with a good sized inverted cone burr followed with a planing with a chisel or hoe) and parallel walls. The walls may be most easily paralleled with a fissure burr. The preparation of the cavity thus far is the same, no matter what may be the material that we use to fill it, but if we are not to use porcelain we will now bevel the cavo-surface angle, for we know that enamel rods that are unsupported will fall out and leave vulnerable places for the recurrence of decay. The great weakness of porcelain lies in the fact that owing to its great friability it is not strong enough to allow the enamel margin to be beveled and thus protected. There are places, however, in the anterior teeth that demand a material that will not be as conspicuous as gold, and then we must resort to porcelain or the silicious cements, which will often give us a filling that has the merit of being inconspicuous and is easily inserted, and has the further advantage over porcelain in that it does not show the black ditch after it has been in for a few months. Unfortunately we can give our patients no assurances of its permanency, and it is, therefore, placed in the category of the temporary expedients, in which class most of us have had to also place porcelain.

The gold inlay is almost ideal for this class of cases in places that are not conspicuous for it can be comparatively easily adapted and with good adaptation as, I believe, a most permanent filling in this position. The cavity preparation is still the same, but now with either the gold filling or the gold inlay, the cavo-surface angle is beveled. This is done with a sharp chisel or hoe and is about the depth of one-quarter the thickness of the enamel; this, when the filling is in place, effectually locks the short enamel rods in the cavity and protects them from falling out.

The cavity outline in all cavities, irrespective of the material used to fill the cavity, must be carried into the areas of relative immunity. This means that the gingival margin must be carried far enough gingivally to have it covered by the healthy gum tissue when the filling is completed, the mesial and distal margins of the cavity must be carried to the angles of the teeth, for it has been determined that the angles are the most invulnerable parts of the tooth surface, and the cavity must be carried far enough incisally, or occlusally, that the food in its excursions over the teeth in mastication will keep this margin clean. It would not be necessary to carry these margins as far as this if we could depend upon our patients keeping their teeth clean, for there is no surface that is more easily kept clean and decay in this surface is usually a pretty good indication that the patient is not as careful as he should be with his tooth brush.

The poorest material that we can choose for these cavities is amalgam. I do not wish to decry amalgam, for I believe that it has saved countless numbers of teeth that would have been otherwise given up to the forceps, but in this position I believe it has a very small place, because the position of the cavity prevents the proper manipulation of the material that is imperative to its successful use. Amalgam to make a good filling must be thoroughly condensed and must be placed in the cavity that will permit of such condensation. If the cavity is not of such shape, as it is not in all proximo-occlusal cavities, we can make it such by the use of the matrix, but in the cavities occurring in the gingival surfaces of teeth, a matrix is not possible and the cavities are necessarily so shallow that any degree of force placed upon the material will tend to displace it at some other part of the cavity. It is for this reason that the cavities that are filled with amalgam in this region so frequently fail.

The finished filling, of whatever material, should be perfectly smooth, for at this portion of the tooth we demand the most perfect finish by reason of the fact that the gingival portion should extend under the free margin of the gum, and if this margin, at least, is not perfectly smooth the delicate gum tissue will become irritated by reason of the rough filling in contact with it and all sorts of trouble will be the result. The gum may retract and leave the margin free to decay again which will be the least of the difficulties that are liable to occur, or the irritation may proceed to an inflammation and that to degenerate into a pyorrhea and the ultimate loss of the tooth. This result has followed so frequently that the mere mention of it is sufficient. Less frequently, the irritation may develop a tumor of the gingivae which may be benign and only manifest its presence by a growth and profuse bleeding upon the slightest touch, or it may in certain cases develop into malignant growths with all of their attendant horrors.

The fact that so many of these fillings are not properly finished is the reason that I am devoting so much time to the consideration of this part of the subject, and I hope that no man in this presence will ever let a patient go again that has an imperfectly finished filling in this neighborhood. A gold filling is somewhat hard to finish at the gingival margin but with proper instruments this difficulty is reduced to the minimum. The Black or Wedelstaedt gold trimmers offer means of reaching these margins and perfectly removing all of the surplus, when the rest of the finish is easily accomplished in the usual manner.

To summarize: The gingival third offers a field of pathological disturbances equaled by no other surface of the tooth.

The frequent sensitiveness met with in patients that have suffered an illness, or particularly, a severe nervous breakdown, calls for immediate relief, and the means are at our command.

Gold is the most permanent material that we can use, but we must be guided in our selection of a filling material by other considerations, and we should use the one that a study of the conditions present indicate.

The cavity preparation for all materials is practically the same with the exception that with porcelain the beveling of the cavo-surface angle is not permissible.

Amalgam is the poorest material to use in this position.

The perfect finish of the filling, particularly the margin extending under the free margin of the gum, is demanded, and the unhappy results of a failure to do so are pointed out.—*Western Dental Journal.*.

